# A Russian Ice Palace

**Published:** 1883-01

**Authors:** 


### A RUSSIAN ICE PALACE.

The idea of the Montreal ice palace is not a new one. Nearly one hundred and fifty years ago its prototype was erected at the whim of the Empress Anne, who reigned from 1730 to 1740. One of the nobles, Prince Galitzin, having changed his religion, was punished by being made a court page and buffoon. His wife being dead, the empress required him to marry again, agreeing to defray the expense of the wedding herself. The prince, true to his new character, selected a girl of low birth. This was in the winter of 1739–40, which was one of extraordinary severity. By her majesty's command, a house was built entirely of ice. It consisted of two rooms, and all the furniture, even to the bedstead, was made of the same material. Four small cannons and two mortars, also of ice, were placed in front of the house and were fired several times without bursting, small wooden grenades being thrown from the mortars. On the wedding-day a procession was formed, composed of more than three hundred persons of both sexes, whom the empress—desirous of seeing how many different kinds of inhabitants there were in her vast dominions—had caused the governors of the various provinces to send to St. Petersburg. The bride and bridegroom were conspicuously placed in a great iron cage, on the back of an elephant. Of the guests (all of whom were dressed in the costume of their respective countries), some were mounted on camels, others were in sledges—a man and woman in each—drawn by beasts of all descriptions, as reindeer, oxen, goats, dogs, hogs and the like. After passing before the imperial palace, and marching through the principal streets of the city, the motley cavalcade proceeded to the Duke of Courland's riding house, where dinner was served to each after the manner of cookery in his own country. The feast over, there was a ball, those from each nation having their own music and their own style of dancing. When the ball was ended, the newly-married pair were conducted to their palace of ice, and guards were stationed at the door to prevent their going out until morning. The building is said to have lasted uninjured, in that cold climate, for several months.

No forest fell<br>

When thou would'st build; no quarry sent its stores<br>

To enrich thy walls; but thou did'st hew the floods,<br>

And make thy marble of the glassy wave.

COWPER.

---

GLOVES are going out of fashion for ladies in Paris. Men left off wearing them years ago.

